# Practical Remarks upon Indigestion, Particularly as Connected with Bilious and Nervous Affections of the Head and Other Parts

**Published:** 1826-01

**Authors:** 


*Practical Remarks upon Indigestion: particularly as connected with Bilious and Nervous Affections of the Head, and other Parts; including Observations upon the Disorders and Diseases of the Stomach, and superior Parts of the Alimentary Canal. Illustrated by Cases.* By JOHN HOWSHIP, Assistant Surgeon to the St. George's Infirmary, &c. &c. 8vo. pp. 204. London, 1825.

PULMONARY consumption has entirely lost its terrors in this country, and INDIGESTION is the order of the day. Nothing is now more common (if we except half-cured syphilis) than the sight of poor wretches wasted to the bones, and spitting large quantities of purulent matter, flattering themselves, and flattered by their medical attendants, that they are just labouring under a little sympathetic cough, and affection of the mucous membrane of the lungs from indigestion! An examination of two minutes, by any man not mounted on a chylopoietic hobby, will prove that, in such cases, there is not a fourth part of the respiratory apparatus left free from disorganization by tubercles, vomicæ, hepatization, &c.! Now, although we do not say that such mistakes produce any material waste of human life, since the undetected disease is generally incurable; yet we do maintain that, in this way, much scandal and discredit are brought upon the medical profession, by errors of diagnosis which a surgeon's apprentice would be ashamed of making. Unfortunately in medicine, as well as in other things, there is generally some reigning error or delusion, which appears to be epidemic for a season, and then gives way to some other fashionable folly. "The *gastro-enterite*" of the French, and the *disorder of the digestive organs* of John Bull, are recent, and, indeed, existing examples.

Some men must necessarily be more instrumental than others, in originating and propagating these theoretical epidemics. Indeed, there is usually a grand master, a strong phalanx of enthusiastic disciples, and a whole host of blind and implicit followers at work, in such periods of popular hallucination. But we have nothing to do with individuals, their objects, their opinions, or their practices. Our observations are directed to prevailing tenets, not to private practitioners—to books, and not to men.

Nor will it be suspected that we underrate the influence of disordered digestion on various other functions of the living machine. None have laboured more zealously, however unprofitably, than ourselves to shew the extent of disorder resulting from such a source. But we cannot shut our eyes to the extremes into which men every day rush, in attributing all diseases to derangement of the chylopoietic viscera. These observations have suggested themselves to us rather from the title than the text of the work before us. In it there is, in fact, extremely little of theory, the work being a collection of facts and observations that have arisen out of Mr. Howship's own practice, and his researches among the records of our art. The work is, indeed, very different from many of the demi-quackeries of the day, entitled treatises on indigestion. It consists of a series of facts and observations on the functional and organic disease of the whole alimentary tube, from the mouth to the anus, and contains but a small number of pages on what is known by the term INDIGESTION.

The first part of the volume is dedicated to affections of the fauces and œsophagus, and treats successively of relaxation of the uvula, enlargement of the tonsils, cynanche, tumours behind the lining of the fauces and pharynx, ulcerated sore throat, ulcerated œsophagus, paralysis of the same, polypous tumours in ditto, pouches in the lining membrane of this tube, spasm of the muscular fibres of the œsophagus, stricture of the same, scirrhus of ditto, &c. From the descriptive portion of this division of the work, we shall introduce a single quotation as a specimen.

"35. Hysterical females are particularly subject to spasmodic constrictions in the œsophagus, generally most troublesome when the stomach is most distressed by flatulence; and while the stomach is struggling for relief by the expulsion of its elastic contents, some part of that canal, rendered more irritable by its sympathy with the stomach, is seized with powerful spasm. The lower parts of the œsophagus, thus violently oppressed by flatulent distension, give the patient the impression, usually compared to that of a large ball moving up and down, threatening suffocation. Under these circumstances, should the patient's health and strength have been previously reduced, we know, upon the authority of Dr. Hooper, that the complaint, from the extreme exhaustion consequent to its continuance, may terminate fatally; although such an event is extremely rare.

"36. Some time since, I had an opportunity of witnessing a most unusual degree of this kind of difficulty and distress, in a thin, middle-aged woman. Somewhat exhausted by walking, when I saw her, a severe paroxysm of aggravated distress came on. The extreme distention of stomach, raising the whole abdomen, almost prevented her drawing any air into the lungs. The rapidity with which the gas was formed almost exceeds credibility. The air, escaping from the stomach, was generally expelled in a forcible loud rush, which continued for the space of half a minute, liberating, perhaps, a volume equal to several pints. She was then able to breathe again, till in a minute, or a minute and a half, the same imperious necessity for breaking off more wind returned as before. During the paroxysm, the quantity of air evolved, judging from the successive volumes set at liberty, must have been immense; as, for near an hour, the average that rushed up from the stomach was, I conceive, at least, equal to a pint every two minutes. As long, however, as the œsophagus remained passive, the distress was comparatively small; but now and then the canal was closed by spasm, preventing the escape of the flatus collected below, and thus occasioning a struggle, severe beyond description, attended with all the convulsive appearances of actual suffocation for the space of nearly half a minute; and, even when relieved by the spasm giving way, being instantly succeeded by a long and continued rush of air from the stomach, the relief to the breathing was scarcely perceptible." 15.

The second part of Mr. Howship's work opens with a slight but spirited sketch of the organ and function of digestion, in the long chain of animated beings, that extends from the hydatid, which is *all stomach*, up to the alderman, who has a few fins and a head superadded. We need hardly say that it would be out of place for us to introduce any part of this anatomico-physiological digression (if we may apply that term to it) into our Journal, leaving it for the public to decide whether it was absolutely called for in a work on indigestion.

From the following passage, we conclude that Mr. H. views the cause of sea-sickness as an affection of the sensorium. We have long been of this opinion, a strong confirmation of which is, the relief obtained by tying a handkerchief round the eyes, and lying down in a dark place, while the ship is in much agitation. When the vibratory motion of all surrounding things is thus excluded from the sight, the sea-sickness becomes greatly abated, as will be found by any one who makes the trial.

"The healthy functions of the stomach are liable to sympathetic disturbance under many affections of the brain. As one of the slightest instances of this kind, I conceive the distressing sense of confusion in the head, and consequent vomiting, in sea-sickness, may be mentioned. In protracted cases of this sort, I have seen the distressing nervous symptoms continue several months, with almost total suspension of the digestive powers; while the frequent straining has repeatedly lacerated the inner membrane of the fauces, inducing hæmorrhage; the disorder eventually leaving the patient little else than a skeleton. Yet, upon reaching shore, the head relieved from its indescribable feelings of distress, the stomach though weak became tranquil, and its powers were re-established. This was the case with a gentleman advised to take a sea voyage for the cure of a spitting of blood; and it certainly did cure it, although by a process his physicians little calculated upon.

The long continuance of sickness, and consequent want of support, soon reducing the vascular system to so low an ebb, as effectually cured the complaint; the physical powers being at the same time so depressed, that any movement of the body induced cramps and spasms even in the abdominal muscles. The bleeding vessel in the lungs, however, closed; and never afterwards gave way." 59.

The above case proves (as far as one instance can prove) another position which we have often advanced, namely, that there is much less danger to the head and lungs from the action of vomiting, than is generally believed. This said action throws the blood so much to the periphery of the body as to greatly secure all the internal vessels from turgescence and rupture.

It is well known that physical affections of the brain very generally derange the digestive function of the stomach. This is properly insisted on by Mr. Howship, but he seems to have overlooked the long list of *moral* affections and emotions of the organ of the mind, which are ten times more operative than the physical lesions above-mentioned, in deranging the functions of the whole chylopoietic viscera.

After enumerating many of the sympathetic disorders of other organs, produced in turn by affections of the digestive apparatus, Mr. Howship touches on the subject of delirium tremens, and from the following passage our author appears to think that he has detected the pathological nature of this obscure disease.

"In cases where, from intemperance, the loss of energy in the stomach has led to ill consequences more immediately depending on the habitually excessive excitement of the nervous powers, delirium of a peculiar character, and oppressed brain from a peculiar cause, progressively ensue. Under these circumstances, also, if the complaints are not cured, the event is fatal. But what do we learn by diligent examination after death? that the ultimate consequence of long suspension of the digestive process, is an absolute deficiency in the necessary supplies of nutritious matter to the vascular system; and that, instead of the proper constituents of healthy blood, the vessels, large and small, especially those within the head, contain a fluid which, in the transparent veins of the brain, appears like water scarcely tinged, and in the arteries is pale and thin, from the great deficiency in the quality and quantity of crassamentum. (Cases 14, 15, 16, 17).

"In a most interesting paper by Dr. Armstrong, of Sunderland,* under the title of brain fever, the peculiar state of brain that exists in this complaint is accurately described, which, by disturbance and oppression of its functions, probably becomes a means of keeping up the extreme debility of stomach, as well as of most other parts of the body. At the conclusion of his valuable remarks, D. A. laments

* Mr. H. might surely have said Dr. Armstrong of LONDON.—*Rev.*

with laudable zeal that repeated solicitation (in a fatal case) failed in obtaining permission to examine the head after death; persuaded that dissection alone can fully determine whether this disease admits of any material variety of treatment.* I hold myself fortunate, that from the kind attention of friends, and other circumstances, I have had repeated opportunities for ascertaining with precision, the condition of the brain, and other viscera, when affected from this cause; and have thus been enabled, I trust, to throw some useful light upon this important part of pathology." 69.

Before offering any remarks on this pathology, we shall furnish our readers with the leading facts of the cases alluded to by our author.

*Case* 1. A publican, aged 40, had been addicted to the use, or rather abuse of ardent spirits for some years. When Mr. H. saw him (May 2nd, 1820) he complained of total loss of appetite, confusion of mind, and evinced occasional sallies of delirium, with extreme irritability, white tongue, and rapid pulse. Mr. H. was informed that, in former attacks of this kind, the patient had been actively treated by bleeding, blistering, &c. notwithstanding which, he was obliged to be confined with a straight-waistcoat for three months, "in a state of insanity." Mr. H. restricted him, at present, to low diet, and exhibited opiates. In the course of three weeks, the patient had greatly mended, and was able to attend to his business. In the following November, Mr. H. was again called to him, and found him with great pain in his head—sleep sometimes overpowering, sometimes entirely absent for nights together—constant coldness of the legs—livid and puffy œdema of the left hand—great irritability of the stomach. The plan before pursued, was again tried; but did not now succeed. Aperients were prescribed. In a few days the patient was found delirious, and wandering about the house all night; but was greatly relieved by an opiate. He went on again for nearly a year, when, having relapsed into a state of inebriety, he died suddenly. Permission was not obtained to examine the body.

*Case* 2. Nov. 12, 1818, Mr. Howship was requested by Mr. Barrow to open the body of a robust man, aged 51 years, who had been long addicted to excessive drinking. In the preceding June he had totally lost his appetite, became very irritable, and highly delirious. He was bled largely, but without relief. By temperance and opiates he was soon res-

" * Edinburgh Medical Journal, vol. ix."

tored. On the 6th of November Mr. Barrow was again summoned. He found that the patient had been cupped, but without benefit. He had been indulging freely. He was desired to live low, and was ordered opiates at short intervals. On the 7th, he was visited by Sir W. K. in consultation, who, suspecting him to be inclined to apoplexy, directed him to be bled to xx. ounces, after which he became more tranquil; but the pulse became weaker, and in the afternoon he was worse in every respect. He was directed to take half a grain of opium every six hours. The following night he was so ungovernably delirious that it required six men to hold him.

"*November 9th.* When the physician saw him, he said "we have here an accession of phrenitis." The head was accordingly directed to be shaved—a blister laid on, and cold lotions applied to the forehead. During the afternoon he was quieter than before; but at 6 p.m. he died."

"*November 12th.* On examination, I found the large veins of the pia mater very full of pale watery blood, and numerous globules of air. There was very little visible character of inflammatory excitement; and only a very sparing serous effusion here and there beneath the arachnoid membrane. In the lateral ventricles near ℥i. of serum was collected on each side; but in structure the brain was perfectly sound.' 171.

*Case* 3. Mr. Howship was requested by Dr. Yeats to examine the body of Capt. G. aged 42 years. This gentleman had been abroad many years, and much addicted to hard drinking, by which his intellects were greatly impaired, and latterly his appetite and general health. A few days before his death he betrayed symptoms of mental derangement, having vehemently abused his servant for breaking a pane of glass, which had never been injured.

"On examination, the tunica arachnoides was found separated from the pia mater, by serous effusion upon the upper, but not the lower, surfaces of the cerebrum. The arachnoid membrane had also numerous small pearly opake spots, upon those parts raised by the effusion of serum. About ℥iij. of a similar fluid was contained in the ventricles. The veins upon the pia mater were loaded with the same peculiarly pale and attenuated fluid, scarcely resembling blood, noticed in my other examinations of this disease.' 172.

*Case* 4. June 7, 1819. Mr. H. was requested to see a kind friend, whose health, from the habit of drinking spirits was nearly destroyed. His appetite was quite lost, and his stomach rejected every thing except "spirits and strongly spiced liquors." From debility and high irritation of system, he had been for many months incapable of attending to business. His memory had failed, and he had strange sensations in his head, with occasional delirium. His skin was dry, tongue covered with a brown fur, pulse small, weak, and 130, bowels relaxed. *A tonic mixture with an opiate. Total abstinence from fermented liquors. June 10th.* Much better. Can eat a little—tongue cleaning fast—pulse down to 90. In the course of a week he resumed his former habits of destructive intemperance, and "soon sunk back from perfect recovery into his former state." His temper sometimes rose to a state of excitement bordering on fury, "at others wandering for weeks under illusions that nothing could dispel regarding the Bank—and a belief, for many months, that he could not stand." One morning he died suddenly, after a restless night.

*Dissection, 27th November*, 1819. A pretty general effusion of serum between the tunica arachnoidea and pia mater, especially towards the basis cranii. The lateral ventricles contained nearly two ounces of blood. The structure of the cerebrum was sound, but that of the cerebellum rather softer than natural. The left vertebral artery was in progress towards ossification. "The blood in the veins upon the pia mater was so thin, as to have the appearance of water slightly tinged with blood, containing, also, large globules of air." The liver was granulated.

We have presented the foregoing cases, as they are considered illustrative of our author's theory of delirium tremens, as stated in the quotation introduced a page or two back.

We confess that, in our apprehension, the cases themselves are very far from being unequivocal examples of delirium tremens; but this we leave to the judgment of the experienced practitioner. In respect to the pathology, as grounded on the *post-mortem* appearances, we have to object that the watery state of the blood in the vessels of the brain is not even a common, much less a constant phenomenon, as far as our own observations have extended. We have seen several instances of exquisitely marked delirium tremens in young men whose digestive organs, up to the accession of the disease, were far from incapable of supplying ample materials for sanguification, and where, on dissection, we found either no particular appearance in the brain, or an unnaturally dry and firm condition of the whole cerebral mass. One of these last kind of cases was examined by us, in the presence of Sir Astley Cooper and some other medical gentlemen, lately. We cannot, therefore, subscribe to Mr. Howship's pathology of this mysterious and dangerous malady. Its true nature is not yet, we think, ascertained. We consider its seat, however, as in the brain and nervous system, rather than in the digestive organs and vascular apparatus. It is a peculiar nervous irritation and irritability, induced by intemperance, and not to be cured by depletion. This is all we know of the disease, and we believe it is all that is known, with any kind of certainty, of it.

Mr. Howship treats of acute and chronic inflammation of the stomach, on which we need not dwell; and, at page 72, comes to the subject of *painful affections* of this organ, probably arising, in many instances at least, from spasm.

"One of the most ordinary, and least serious causes of this complaint, is that state of stomach and bowels connected with habitual costiveness. Some persons are occasionally distressed in this way for very many years; for when suffering more than usual, induced to refer to medicine, they are for a time relieved. I have attended to this complaint at the early age of six years, and have met with it at all subsequent periods of life. In the young it is described as a severe fixed pain, always referred to the same spot, the pit of the stomach, which makes them cry with distress. In those who are older it is compared to the stomach being grasped tight in the hand, or fixed in a vice; with sense of weight, and faintness. In all cases it is connected with dyspepsia, the digestive powers being nearly, or entirely suspended, not only during the paroxysm but during the continuance of any tendency to this disorder. (Case 19.)

"This affection often seems to depend principally on deficient action of bowels; for when stools are procured, it appears that the little taken into the stomach is properly digested, and the residue tinged with healthy looking bile. Sometimes, however, this complaint is evidently connected with morbid secretions from the chylopoietic viscera; the bowels not being merely loaded, but filled with the most unhealthy matters. In both these states, the tongue will generally be either white, or furred; the pulse seldom much disturbed." 73.

The most terrible cases which Mr. Howship has ever seen, have been those which occurred in gouty subjects, and where there was reason to believe the arthritic irritation had been transferred to the stomach from the extremities.

"Upon this point, however, Dr. Hooper has in conversation acquainted me, that he does not conceive any spasmodic affections of stomach, attributable to gout, can be traced after death, as such; for that the stomach in gouty habits is usually flatulent, and very relaxed; and that although a person from retrocedent gout may be seized with violent and terrible pain, referred to the stomach, with vomiting of a coffee-ground-like matter, even proving suddenly fatal, almost as if killed by a cannon-shot, yet the stomach has been found uniformly relaxed, exhibiting only patches of increased capillary vascularity on its internal surface. This he has himself seen in several such cases, and upon mentioning the matter to the late Dr. BAILLIE, he observed, he believed it was so, and that he had seen much the same appearances, and those only. Dr. HOOPER additionally remarked, that the violence of the pain was not positively known to be seated in the stomach, although there was much reason to believe it to be so, for the semilunar ganglion might be its seat; and that where muscular contraction without structural disease is found, it may be considered the natural, or at least the accidental state of the part, unconnected with any effect of disease." 75.

A remarkable case, connected with the present subject, is quoted from Dr. Pye. The patient was a medical man, severely affected with gout in both feet. In the midst of the paroxysm, the pains flew like lightning to the calves of the legs, leaving the feet entirely free. In half a minute they changed their seat, with the same rapidity, to the thighs—and in less than a minute darted to the bowels where they gave the patient one or two twitches, and then ascended to the stomach. Here the pains and the fit of gout ended, upon the patient's vomiting up a pint and a half of green aqueous liquor, so extremely corrosive as to be compared to the strongest mineral acid. He fell asleep immediately after this, and awoke free from complaint. A natural inquiry here follows:—were the spasmodic pains in the muscular structure of the limbs, bowels, and stomach, the cause or the effect of the formation of the above-mentioned fluid? We think there can be no doubt that a depraved secretion in the stomach or bowels will often occasion cramps and spasms in various parts of the body; but whether cramps or spasms can so derange the function of the stomach as to produce a new and peculiarly corrosive secretion there, is not so easily proved, or even rendered probable.

Hepatitis next engages our author's attention, and obtains a short disquisition. Then follows the subject of biliary calculi, and cases are quoted from various authorities; but nothing new is elicited. Splenitis is also treated of by Mr. Howship, and researches made on that subject. Scirrhus, cancer, polypus, hæmorrhage next succeed, and are very cursorily discussed. Two cases are related of swallowing boiling water—in one, the patient's life was saved,—in the other, death took place. In the latter, on examination, there was found inflammation in the stomach as well as in the trachea and œsophagus.

Thus ends the descriptive and pathological portion of the work—there being very little said on what is commonly understood by the term "INDIGESTION." At page 112, the treatment of the various affections above enumerated, is entered upon—and from this we shall be able to select very little, since the directions, though generally judicious, present little that is beyond common place. A single extract will afford an example of the manner and matter, in the therapeutical division of this work.

"That particular variety of dyspepsia (167) which seems to depend on a depraved state of the secretions, from the glandular parts and internal surface of the stomach, I have ventured to distinguish as giving origin to the scrofulous habit, because I have frequently seen them so associated with each other in the same individual, that when attentively considered, it was impossible to draw any other conclusion.

"The unhealthy state of the gastric secretions might in most of these cases be taken for granted, from the permanently defective power of digestion, the irksome and continued sense of local uneasiness, flatulence and acid eructations. This important fact, however, is occasionally proved, by the irritation from these fluids inducing the stomach from time to time to relieve itself by rejecting its contents; the matter thrown up being principally a stiff, ropy, bilious, sour, or corrosive mucous fluid. In one instance to which I paid particular attention, my patient remarked, he always found himself better in warm weather, and that a voyage to the West Indies was sure to exempt him from his complaints until he returned to England.

"As to the treatment best adapted to correct this state of constitution, I have in various instances tried the preparations of steel, the vegetable tonics, mercurials, aperients, and purgatives, without material effect. It is in this particular case that I conceive sarsaparilla calculated to prove an essentially useful medicine; although it is probably necessary to assist its favourable influence, by uniting it with other means. The plan most to be relied upon, is to place the patient upon a course of the compound decoction of sarsaparilla, in combination with the carbonate of soda, or potash, and any aperient medicine. The particular mode of adjusting these medicines to each other, and to the peculiar circumstances of the constitution, must of course differ in every two cases. In some instances, the uneasiness about the stomach is equalled or exceeded by uncomfortable sensations referred to the right side, arising from duodenal irritation and congestion; here, of course, the aperient influence must be carefully maintained. Many illustrations might have been brought forward, of the pathology, and successful treatment of this disorder; but one example distinctly marked has been considered sufficient." 128.

The work concludes with a selection of cases, 29 in number, in illustration of many of the topics touched upon in the text. The great fault of the performance is the attempt to treat of so many different subjects, any one of which would require, for proper investigation, the whole limits of Mr. Howship's volume. On this account the work is rather too cursory and superficial for the professional—and too technical for the popular reader. It contains, however, a great number of interesting facts and observations, which will repay the perusal of the junior branches of the profession.

